# National and regional prevalence of gestational diabetes mellitus in India: a systematic review and Meta-analysis

**DOI:** 10.1186/s12889-024-18024-9

**Published:** 2024-02-20

**Authors:** Neha Mantri, Akhil Dhanesh Goel, Mamta Patel, Pritish Baskaran, Gitashree Dutta, Manoj Kumar Gupta, Vikas Yadav, Madhukar Mittal, Shashank Shekhar, Pankaj Bhardwaj

**Affiliations:** 1 School of Public Health, AIIMS, Jodhpur, India; 2 Department of Community Medicine & Family Medicine, AIIMS, Jodhpur, India; 3 ICMR-NIREH, Bhopal, Madhya Pradesh India; 4 Department of Endocrinology and Metabolism, AIIMS, Jodhpur, India; 5 Department of Obstetrics and Gynaecology, AIIMS, Jodhpur, India; 6 Department of Community Medicine & Family Medicine, Academic Head, School of Public Health, AIIMS, Jodhpur, India

**Keywords:** Gestational diabetes mellitus, Pregnancy complications, Diagnosis, IADPSG, Glucose tolerance test, DIPSI

## Abstract

**Background:**

Gestational diabetes mellitus (GDM) is frequently misdiagnosed during pregnancy. There is an abundance of evidence, but little is known regarding the regional prevalence estimates of GDM in India. This systematic review and meta-analysis aims to provide valuable insights into the national and regional prevalence of GDM among pregnant women in India.

**Methods:**

We conducted an initial article search on PubMed, Scopus, Google Scholar, and ShodhGanga searches to identify quantitative research papers (database inception till 15th June,2022). This review included prevalence studies that estimated the occurrence of GDM across different states in India.

**Results:**

Two independent reviewers completed the screening of 2393 articles, resulting in the identification of 110 articles that met the inclusion criteria, which collectively provided 117 prevalence estimates. Using a pooled estimate calculation (with an Inverse square heterogeneity model), the pooled prevalence of GDM in pregnant women was estimated to be 13%, with a 95% confidence interval (CI) ranging from 9 to 16%.. In India, Diabetes in Pregnancy Study of India (DIPSI) was the most common diagnostic criteria used, followed by International Association of Diabetes and Pregnancy Study Groups (IADPSG) and World Health Organization (WHO) 1999. It was observed that the rural population has slightly less prevalence of GDM at 10.0% [6.0–13.0%, I^2^_=_96%] when compared to the urban population where the prevalence of GDM was 12.0% [9.0–16.0%, I^2^ = 99%].

**Conclusions:**

This review emphasizes the lack of consensus in screening and diagnosing gestational diabetes mellitus (GDM), leading to varied prevalence rates across Indian states. It thoroughly examines the controversies regarding GDM screening by analyzing population characteristics, geographic variations, diagnostic criteria agreement, screening timing, fasting vs. non-fasting approaches, cost-effectiveness, and feasibility, offering valuable recommendations for policy makers. By fostering the implementation of state-wise screening programs, it can contribute to improving maternal and neonatal outcomes and promoting healthier pregnancies across the country.

**Supplementary Information:**

The online version contains supplementary material available at 10.1186/s12889-024-18024-9.

## Background

Manifestation of glucose intolerance in pregnancy, often, named Gestational Diabetes Mellitus (GDM) is emerging as a major public health problem. The World Health Organization 1999 report provides a fundamental definition which states “Gestational diabetes is a carbohydrate intolerance resulting in hyperglycemia of variable severity with onset or first recognition during pregnancy” [1]. Nevertheless, there has been substantial debate over how to characterize glucose in pregnancy, which has complicated clinical work and research over the past three decades. Additionally, it may start at the same time as pregnancy, which increases the risk of it going undetected and having adverse maternal and neonatal complications [2–6].

In 2015, the International Diabetic Federation (IDF) reported that 1 in 11 people worldwide have diabetes, with 75% of them residing in low and middle-income countries [7]. There is a huge variation in the prevalence of GDM globally from 10.1% (Eastern & Southeastern Asia) to 13.61% (Africa) depending on screening strategies, diagnostic criteria, and the background population’s ethnic composition [8, 9]. South East Asia region had 6.9 million live births being affected by hyperglycemia in pregnancy; with an estimated prevalence of 24.2% [10]. India, being the largest populous country in the world, shows the prevalence of GDM in the ranging from 3 to 35% [11–15].

Currently, the Diabetes in Pregnancy Study Group of India advocates for universal screening using a single non-fasting 2-h 75 g OGTT, with 2 h value > 140 mg/dL being diagnostic of GDM [16]. The International Association of Diabetes and Pregnancy Study Groups (IADPSG) criteria are based on the findings of the large-scale Hyperglycemia and Adverse Pregnancy Outcome (HAPO) study and hence popular globally, [17] but its drawback is argued to be the large number of false-positive cases due to lower fasting cutoffs and hence adding to the burden of GDM [18, 19]. In addition, diagnosing the Indian population by international studies can be inconclusive as the HAPO study lacked Indian representativeness in its findings [17].

To solve the inconsistencies in diagnosis and management of GDM, a technical and operational guideline has been developed under the aegis of the Maternal Health Division, Ministry of Health and Family Welfare, Government of India in February 2018 [20]. However, subsequent studies have shown high variability in the prevalence, from rates as low as 0% in Manipur to 42% in Lucknow, Uttar Pradesh [21, 22]. A variety of factors may contribute to this variability, including differences in the genetics and population across India, as well as differences in screening practices.

A pan India prospective study (2021) conducted by FOGSI and DIPSI shows about one-third of the pregnant women are diagnosed with GDM during the first trimester and over a quarter of them have a history of fetal loss in the previous pregnancies [23]. Hence, GDM is a topic of considerable controversy when it comes to its screening, diagnosis and its cost-effectiveness.

With this aim, we conducted a systematic review to estimate the national and regional prevalence of GDM in pregnant women to foster the implementation of programs state-wise effectively. This analysis aims to investigate how various factors, such as different screening criteria, geographical locations (urban versus rural areas), techniques used for blood collection, and the timing of screening during pregnancy (early versus late), might influence the observed prevalence of GDM in pregnant women in India.

## Methodology

### Study protocol

This Systematic Review and Meta-Analysis is written in accordance with the Preferred Reporting Items for Systematic Reviews and Meta-Analysis (PRISMA) guidelines [24] and is registered in the International Prospective Register of Systematic Reviews (PROSPERO) database (Ref.no. CRD42022335011).

### Search strategy

We framed our research question using the PICO(S)(T) methodology (Population-pregnant women; Intervention-nil; Comparison-nil; Outcome-GDM; Study design-cross-sectional in India).

We performed searches in PubMed and Scopus using selected keywords. These results were supplemented by relevant studies from Google Scholar and ShodhGanga—Indian thesis repository (https://shodhganga.inflibnet.ac.in/). The last day fir performing the search was 15th June 2022. No date or language restrictions were imposed. The cross-references of the included studies were explored for additional studies. Keywords were identified by iterative discussion among reviewers, and a search query was developed separately for each database. The controlled descriptors (such as MeSH terms) and Boolean operators were used to develop a robust search strategy. (See Additional file 1: Search Strategy).

### Eligibility criteria

The studies reporting the prevalence of GDM in pregnant women in India were included.

### Inclusion criteria

1. Community or hospital-based studies.
2. Original published articles and short communications.
3. Studies providing the prevalence of GDM
4. Studies conducted in India
5. Type of studies: cross-sectional studies.

### Exclusion criteria

1. Overviews, editorials, other review papers, or method protocols without results
2. Molecular or genetic studies, animal studies, Invitro studies.
3. Studies that did not differentiate between GDM and type 1 and/or type 2 diabetes
4. Studies that reported risk factors, associated biomarkers, or outcomes of GDM without reference to GDM prevalence
5. Studies which have not reported screening methods
6. Experimental studies
7. Three authors independently examined search results for inclusion. Disagreements, if any, were settled by consensus with a fourth author.

### Study selection

A reviewer independently conducted searches on all information sources from various databases and uploaded to Rayyan QCRI online software [25]. Rayyan QCRI helped in ensuring a systematic and comprehensive search and selection process. A fourth reviewer managed Rayyan QCRI software, who identified and removed the duplicate citations. Three authors independently screen titles and abstracts with turned “blind” function on. The discrepancies between the three reviewers were discussed with a fourth author for making a consensus to select the articles. Full-text copies of all selected studies were obtained to find more details. We documented the reasons for the exclusion of studies explored as full text. The study inclusion process is presented using the PRISMA flowchart. The reference management software Mendeley Desktop (https://www.mendeley.com) for Windows was used to store, organize, cite, and manage all the included articles.

### Data extraction

After selecting eligible studies, we obtained full-text articles for all included studies. Two reviewers independently performed data extraction of relevant information. Data were extracted regarding author, year of publication, study location, site (hospital- or community-based or data-based), study type, trimester, sample size, diagnostic criteria, and prevalence of GDM. We recorded investigators’ definitions of GDM and screening and diagnostic criteria used for GDM.

When a study reports the prevalence of GDM in the same population using multiple diagnostic criteria, the most recent and up-to-date criteria was selected in the following sequence-.IADPSG/ WHO 2013 > DIPSI> WHO 1999 > ADA > NICE> Carpenter and Coustan > NDDG> O’Sullivan and Mahan’s Criteria as framed after the iterative discussion.

#### Bias reporting

The methodological quality of the studies was analyzed independently by two investigators using the AXIS tool which critically appraises study design and reporting quality as well as the risk of bias in cross-sectional studies. We assessed bias using the AXIS Tool for Prevalence Studies in our systematic review [26]. The AXIS tool has 20 components assessing the quality of the studies with special focus on the presented methods and results based on a combination of evidence, epidemiological processes, experience of the researchers and Delphi participants. The components included in this checklist are addressing study objective, design, sample size, sample population, sample frame, selection process, non-responders, risk factors and outcome measured, appropriateness of statistical methods, consistency of results, discussion justified, limitation of the study, ethical approval and any conflict of interest or funding received.

#### Data synthesis and analysis

The prevalence of GDM from different studies were pooled together using the Inverse variance heterogeneity method. Heterogeneity was assessed using I^2^ Statistics. High heterogeneity in the study was analyzed using sub-group analysis and sensitivity analysis. MetaXL software was used for data synthesis [27]. Publication bias was determined using DoI plot and LFK index.

## Results

**Fig. 1:**
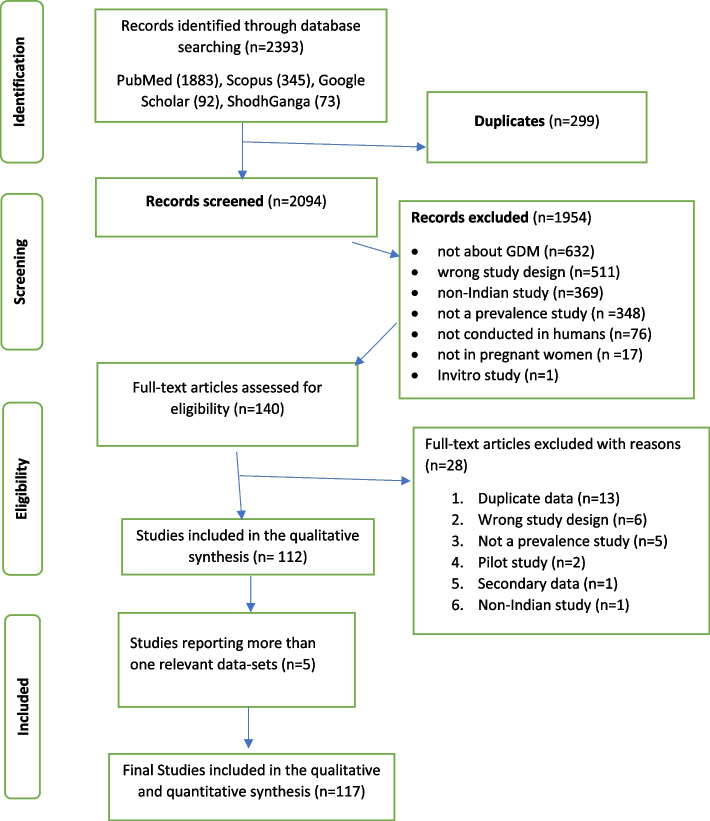
PRISMA Flowchart

On searching PubMed (*n* = 1883), Scopus (*n* = 345), Google Scholar (*n* = 92), and ShodhGanga—reservoir of Indian theses (*n* = 73), a total of 2393 articles were identified related to GDM (see Fig. 1: PRISMA flowchart) Thus, the full texts of 140 articles were assessed for eligibility. During this process, a total of 13 authors were contacted for full-text via email, out of which (*n* = 11) responded. Remaining 2 articles were included based on only abstract and in data extraction sheet, missing data were reported. Thus, a final 117 articles were included in the systematic review and meta-analysis for the analysis. (See Table 1: Data Extraction Sheet).

**Table 1:** Data Extraction Sheet

S.No	Author/Year	City/State	Study Setting	Scale	Participant characteristics	prevalence
1	Panigrahi A et al. 2020	Bhubaneshwar (Orissa)	Hospital-based study Urban Duration = 2015–2016	DIPSI	N = 30 Gestational Age = 24 to 32 weeks Mean Age = 31.30 ± 4.17 years	13.8%
2	Seshiah V et al. 2008	Chennai (Tamil Nadu)	Hospital-based study Urban	WHO 1999	*N* = 87 Mean age = 28.38 ± 4.31 yrs	42.03%
3	Suchitra M.R. et al. 2020	Kumbakonam (South India)	Hospital-based study Semi-urban Duration = Oct.2018 to Nov.2018	WHO 1999	N = 10 Gestational Age = 24 weeks Age = 26.6 years	3.8%
4	Chandramathy K et al. 2018	Calicut (Kerala)	Hospital-based study Semi-urban Duration = November 2009 to April 2010.	WHO 1999	*N* = 33 Gestational Age = 20 to 28 weeks Age = 24 years	6.9%
5	Agarwal S et al. 2018	Lucknow (Uttar Pradesh)	Hospital-based study Urban Duration = Januray 2016 to December 2016	DIPSI	*N* = 814 Gestational Age = 24 to 28 weeks	13.9%
6	Patel M et al. 2018	Lucknow (Uttar Pradesh)	Hospital-based study Urban Duration = 1 Year	DIPSI	*N* = 351 Gestational age = 24 to 28 weeks	3.91%
7	Kumar N et al. 2018	Lucknow (Uttar Pradesh)	Hospital-based study Urban Duration = Jan 2016 to Jan 2017	DIPSI	*N* = 209 Gestational Age = 24 to 28 weeks Age = 19 to 42 years	10.9%
8	Sharma M et al. 2018	Delhi	Hospital-based study Urban Duration = June 2014 to May 2016	IADPSG	*N* = 16 Gestational Age = less than 20 weeks Mean Age = 24.56 ± 2.87	6.5%
9	Trivedi D. et al. 2017	Ahmedabad (Gujarat)	Hospital-based study Urban Duration = April 2014 to April 2016	DIPSI	*N* = 23 Gestational Age = 24 to 28 weeks	10.95%
10	Satyajit P G et. 2017	(Loni) Maharashtra	Hospital-based study Rural Duration = 15th September 2014 to 14th September 2016.	DIPSI	*N* = 26 Gestational Age = 24 to 28 weeks	5.20%
11	Goswami Mohanta et al. 2014	Dibrugarh (Assam)	Hospital-based study Rural Duration = June to August 2011	WHO 1999	*N* = 28 Gestational Age = in the first trimester Mean Age = 22.56 ± 4.532 years	3%
12	Sawant A P et al. 2011	Sai Shirdi (Maharashtra)	Hospital-based study Rural	WHO 1999	N = 18 Gestational Age = 14–16 weeks	3.6%
13	Surapaneni T et al. 2010	Hyderabad (Andhra Pradesh)	Hospital-based study Urban Duration = Jan 2007- Dec. 2007	WHO 1999	*N* = 270 Gestational Age = 1st trimester Mean Age = 26.07 ± 4.23 years	8.43%
14	Saxena P et al. 2022	New Delhi	Hospital-based study Urban	DIPSI	*N* = 107 Gestational Age = 24 to 32 weeks	10.4%
15	Tripathi R et al. 2022	New Delhi	Hospital-based study Urban Duration = 1 year	IADPSG	N = 201 Gestational Age = 24 to 28 weeks	8.8%
16	Shah C S et al. 2022	Karamsad, Anand District (Gujarat)	Hospital-based study Rural Duration = Feb 2019- May 2020	DIPSI	*N* = 52 Gestational Age = 24 to 28 weeks Age = 21 to 30 years	17.33%
17	Chebrolu P et al. 2021	Chattisgarh	Community-based study Rural Duration = March 2017 to April 2018	DIPSI	N = 11 Gestational Age = 24 to 28 weeks Mean Age = 26 years	1.9%
18	Deepa R et al. 2020	Bengaluru	Hospital-based study Urban Duration = April 2016 to September 2018	WHO 1999	*N* = 313 Gestational Age < 36 weeks Age = 18 to 45 years	17.6%
19	Chanda S et al. 2020	Assam	Community-based study Rural Duration = July 2019 to September 2019	WHO 2013	*N* = 202 Gestational Age = 24 to 28 weeks Mean Age = 23.7 years (SD ± 4.20)	16.67%
20	Hussain T et al. 2020	Bhubaneshwar	Hospital-based study Urban	WHO 2013	*N* = 154 Gestational Age = 24 to 32 weeks Age = 18–25 years (57%) 26–33 years (33%)	9.89%
21	Todi S et al. 2020	Puducherry (South India)	Hospital-based study Urban Duration = March 2017 to October 2018	IADPSG	*N* = 185 Gestational Age = upto 34 weeks Mean Age = 26.02 years	25.1%
22	Taneja A et al. 2020 (A)	Punjab	Hospital-based study Urban Duration = Jan 1 to Dec 31, 2015	?	N = 7 Gestational Age = 26 to 28 weeks	6.6%
23	Taneja A et al. 2020 (B)	Punjab	Hospital-based study Urban Duration = Jan 1 to Dec 31, 2015	?	N = 11 Gestational age = after 34 weeks	13%
24	Chaudhry M et al. 2019	Belgavi (Karnataka)	Hospital-based Study Rural Duration = Jan 2016 to August 2017	DIPSI	*N* = 69 Gestational Age < 20 weeks	16.1%
25	Chudasama R K et al. 2019	Rajkot (Gujarat)	Hospital-based study Urban Duration = Jan to March 2016	WHO 2013	*N* = 41 Gestational Age = 21 to 28 weeks Age = 21 to 25 years	11.5%
26	Rajasekar G et al. 2019	Vellore (Southern India)	Hospital-based study Rural Duration = February to July 2015	IADPSG	N = 88 Gestational Age = 24 to 28 weeks Mean Age = 25.27 ± 4.42 years	14%
27	Dubey D et al. 2019	Lucknow (Uttar Pradesh)	Hospital-based study Urban	WHO 1999	*N* = 51 Gestational Age = 24 to 28 weeks	19.6%
28	Nachankar A et al. 2018	Delhi Cantt (New Delhi)	Hospital-based study Urban Duration = Dec 2016 to June 2017	ADA	N = 14 Gestational Age = 24–28 w Mean Age = 27.5 years ±2.9 years	18.7%
29	Agarwal M et al. 2018	New Delhi	Hospital-based study Urban Duration = 01 Jan 2013 to 31 Dec 2015	IADPSG	*N* = 1193 Gestational Age = 24 to 28 weeks Mean Age = 27.4 ± 3.9 years	18.3%
30	Saxena P et al. 2017	New Delhi	Hospital-based study Urban	DIPSI	N = 63 Gestational Age = 24 to 32 weeks Mean Age = 27.98 ± 4.3 years	7.87%
31	Tripathi R et al. 2017	New Delhi	Hospital-based study Urban Duration = Oct 2011 to Feb 2013	IADPSG	*N* = 64 Gestational Age = 24–28 weeks	6.8%
32	Tahmina S et al. 2017	Pondicherry	Hospital-based study Urban Duration = Apr 2013-March 2014	IADPSG	*N* = 167	22.78%
33	Singh A et al. 2016	Raipur (Chhattisgarh)	Hospital-based study Urban	WHO 2013	*N* = 156 Gestational Age = 24 to 28 weeks Mean Age = 25 to 29 years	5.2%
34	Bhavdharini et al. 2016 (WINGS 6) (A)	Chennai (Tamil Nadu)	Hospital-based study Urban Duration = Jan 2013 to Dec 2014	IADPSG	*N* = 210 Gestational age = All trimester Mean Age = 26.5 ± 4.2 years	16.1%
	Bhavdharini et al. 2016 (WINGS 6) (B)	Chennai (Tamil Nadu)	Hospital-based study Rural Duration = Jan 2013 to Dec 2014	IADPSG	*N* = 68 Gestational age = All trimester Mean Age = 26.5 ± 4.2 years	14.4%
35	Mohan M A et al. 2016	Kerala	Hospital-based study Urban Duration = January to October 2014	WHO 1999	*N* = 32 Gestational Age > 24 weeks Mean age = 28.53 4.76 years	15.9%
36	Kragelund Nielsen K et al. 2016 (A)	Tamilnadu	Hospital-based study (Health centers) Rural Duration = June 2012 to July 2014	DIPSI	N = 30 Gestational Age = All trimester Mean Age = 26.8 (4.5) years	8.0%
	Kragelund Nielsen K et al. 2016 (B)	Tamilnadu	Hospital-based study (Health centers) Semi-urban Duration = June 2012 to July 2014	DIPSI	*N* = 385 Gestational Age = All trimester Mean Age = 26.8 (4.5) years	13.3%
	Kragelund Nielsen K et al. 2016 (C)	Tamilnadu	Hospital-based study (Health centers) Urban Duration = June 2012 to July 2014	DIPSI	*N* = 244 Gestational Age = All trimester Mean Age = 26.8 (4.5) years	30.7%
37	Veeraswamy S et al. 2016	Tamil Nadu	Hospital-based study Urban Duration = August 2013 to December 2013	WHO 2013	*N* = 740 Gestational Age = All trimester	8%
38	Soumya S et al. 2015	Chandigarh	Hospital-based study Urban	IADPSG	*N* = 45 Gestational Age = 24 to 28 weeks	9%
39	Arora G P et al. 2015 (A)	Punjab (North India)	Hospital-based study Urban Duration = August 2009 to December 2012	WHO 2013	*N* = 458 Gestational Age = 24 to 28 weeks Mean Age = 21.7 ± 3.4 years	9%
40	Arora G P et al. 2015 (B)	Punjab (North India)	Hospital-based study Rural Duration = August 2009 to December 2012	WHO 2013	*N* = 1779 Gestational Age = 24 to 28 weeks Mean Age = 21.7 ± 3.4 year	34.9%
41	Bhatt AA et al. 2015	Pune (Maharashtra)	Community-based study Rural Duration = Sep. 2012 to June 2014	DIPSI	*N* = 94 Gestational Age < 24 weeks Mean Age = 22.7 ± 3.1 years	9.5%
42	Gopalakrishnan V et al. 2015	Lucknow (Uttar Pradesh)	Hospital-based study Urban Duration =July 2012 to July 2013	IADPSG	*N* = 139 Gestational Age = 24 to 28 weeks Mean Age = 25.1 ± 3.9 years	41.9%
43	Dave V R et al. 2014	Gujarat	Community-based study Rural Duration = March 2013 to June 2013	ADA	N = 6 Gestational Age = All trimester Mean age=	1.73%
44	Mohan V et al. 2014	TamilNadu (Chennai)	Hospital-based study Urban and Rural Duration = Jan 2013 to Nov 2013	IADPSG	N = 52 Gestational Age = All trimester Mean Age = 24 ± 3.1 years	5.04%
45	Rajput M et al. 2014	Rohtak (Haryana)	Community-based study Rural	WHO 1999	*N* = 127 Gestational Age > 24 weeks Mean Age = 24.0 ± 3.1 years	13.9%
46	Neelakandan R et al. 2014	Tiruchiraalli (TamilNadu)	Hospital-based study Urban Duration = Feb 2012 to Jan 2013	IADPSG	*N* = 258 Gestational Age = all trimester	23.3%
47	Vanlalhruaii et al. 2013	Imphal (Manipur)	Hospital-based study Semi-urban Duration = September 2010 to August 2012	ADA	*N* = 37 Gestational Age = 24 to 28 weeks	12.33%
48	Surapaneni T et al. 2013	Hyderabad	Hospital-based study Urban	IADPSG	*N* = 520 Gestational Age = 24 to 28 weeks Mean age = 27.18 (3.95) years	21.81%
49	Sharma K et al. 2013	Jammu	Hospital-based study Urban Duration = October 2010 to September 2011	WHO 2013	*N* = 55 Gestational Age = 16 to 32 weeks Mean Age = 30 years	11%
50	Kalra P et al. 2013	Jodhpur (Rajasthan)	Hospital-based study Urban Duration=	DIPSI	N = 33 Gestational Age = 24 to 28 weeks Mean age = 25.33 ± 3.17 years	6.6%
51	Ghosh S et al. 2013	Kolkata	Hospital-based study Urban Duration = July 2009 to June 2010	?	*N* = 58 Gestational Age = Any trimester Mean Age = 30 years	9%
52	Rajput R et al. 2013	Haryana	Hospital-based study Urban Duration = June 2009 to January 2011	ADA	*N* = 43 Gestational age = 24 to 28 weeks Mean Age = 23.62 ± 3.42 yr	7.1%
53	Seshiah V et al. 2012	Chennai	Hospital-based study Urban	IADPSG	*N* = 214 Gestational Age = Any trimester	14.6%
54	Dwarkanath P et al. 2019	Bangalore	Hospital based study Urban Duration = 2008 to 2014	IADPSG	*N* = 392 Gestational Age = 24 to 28 weeks	10.2%
55	Grewal E et al. 2012	New Delhi	Hospital-based study Urban Duration = July 2006 to Jan 2009	Carpantan and Coustan Criteria	*N* = 46 Gestational Age = 24th -28th weeks Mean age = 26.87 ± 4.0 years	15.49%
56	Tripathi R et al. 2011	New Delhi	Hospital-based study Urban	Carpantan and Coustan Criteria	N = 74 Gestational Age = 24 to 28 weeks Mean Age = 25.9 ± 4.4 years	10.8%
57	Balaji V et al. 2012	Chennai	Hospital-based study (Therapeutic center) Urban	WHO 1999	*N* = 86 Gestational Age = Third trimester Mean Age = 23.8–3.48 years	10.5%
58	Somani B et al. 2012	Pune (Maharashtra)	Hospital-based study Urban	WHO 1999	N = 35 Gestational Age = 24 to 28 weeks Mean Age = 23.45 years	4.8%
59	Jali M V et al. 2011	Belgaum (Karnataka)	Hospital-based study Urban Duration = May 2008 to April 2010	WHO 1999	N = 52 Gestational Age = 24 to 28 weeks	16%
60	Balaji V et al. 2011	Chennai (Tamil Nadu)	Hospital-based study Urban Duration = April 2009 to February 2010	IADPSG	*N* = 1463 Gestational Age = second and third trimester Mean Age = 23.6 ± 3.3 years	3.2%
61	Wahi P et al. 2011	Jammu	Hospital-based study Semi-urban Duration = December 2007 to November 2008	DIPSI	*N* = 2025 Gestational Age = 24 to 28th week Mean Age = 27.2 ± 2.3 years	6.51%
62	Seshiah V et al. 2009 (A)	Chennai city (Tamil Nadu)	Community-based study Urban Duration = 2005–2007	WHO 1999	*N* = 739 Gestational Age = 24 to 32nd week Mean Age= 23.7 ± 3.55 years	17.8%
63	Seshiah V et al. 2009 (B)	Saidapet (Tamil Nadu)	Community-based study Semi-urban Duration = 2005–2007	WHO 1999	*N* = 548 Gestational Age = 24 to 32nd week Mean Age = 23.4 ± 3.30 years	13.8%
64	Seshiah V et al. 2009 (C)	Thiruvallur (Tamil Nadu)	Community-based study Rural Duration = 2005–2007	WHO 1999	N = 392 Gestational Age = 24 to 32nd week Mean Age = 22.5 ± 3.09 years	9.9%
65	Krishnaveni GV et al. 2007	Mysore (Karnataka)	Hospital-based study Urban Duration = 1997–98	WHO 1999	N = 35 Gestational Age = 30–32 weeks	6.65%
66	Seshiah V et al. 2007	Chennai (Tamil Nadu)	Hospital-based study Urban	WHO 1999	*N* = 741 Gestational Age = 24–28 weeks Mean age = 23.66 ± 3.55 years	17.9%
67	Wani AI et al. 2005	Kashmir	Hospital-based study Urban	WHO 1999	*N* = 198 Gestational age = second and third trimester	4.4%
68	Hill JC et al. 2005	Mysore (Karnataka)	Hospital-based study Urban Duration = June 1997–August 1998	Carpantan and Coustan Criteria	*N* = 49 Gestational age = 30 + 2 weeks Mean age = 23.6 years	6.2%
69	Seshiah V et al. 2004	Chennai (Tamil Nadu)	Hospital-based study Urban	WHO 1999	*N* = 168 Gestational age = Second and third trimester Mean age = 23 ± 4 years	18.9%
70	Bhattacharya SM 2004	Kolkata (West Bengal)	Hospital-based study Urban	Carpantan and Coustan Criteria	N = 26 Gestational age = 24–28 weeks	10.5%
71	Ramachandran A et al. 1994	Chennai (South India)	Hospital-based study Urban Duration = September to December 1992	O’Sullivan and Mahan’s criteria	N = 4 Gestational age > 24 weeks Mean age = 29.3 ± 2.5 years	0.56%
72	Pal A et 2018	Shimla (Himachal Pradesh)	Hospital-based study Urban Duration = 1st August 2014 to 31st July 2015	DIPSI	N = 30 Gestational age = 24–28 weeks	6%
73	Vidya M sree et al. 2020	Chennai	Hospital-based study Urban Duration = Jan 2019 to Dec 2019	DIPSI	*N* = 136 Gestational age = 24–28 week Mean age = 26.09 years	13.6%
74	Mounika E et al. 2018	Karimnagar (Telangana)	Hospital-based study Urban	IADPSG	*N* = 40 Gestational Age = 24–28 weeks	6.67%
75	Naik RR et al. 2019	Goa	Hospital-based study Urban Duration = Nov 2014 to April 2016	Carpantan and Coustan Criteria	*N* = 424 Gestational age = 24 to 28 weeks Mean age = 31.2 years	5.49%
76	Dwarkanath Let al 2019	Tumkur	Hospital-based study Urban Duration = August 2014 to Oct 2016	DIPSI	N = 7 Gestational age = 24–28 weeks Mean age=	3.5%
77	Muthuramalingam V et al. 2020	Tamil nadu	Hospital-based study Rural Duration = Nov 2016 to Dec 2019	WHO 2013	N = 94 Gestational age = 24–28 weeks Mean age = 27.54 ± 3.58 years	16.06%
78	Balagopalan al 2021	New Delhi	Community-based study Urban and Rural Duration = Dec 2017 to Dec 2018	IADPSG	*N* = 138 Gestational age = 18–28 weeks	27.3%
79	Jadhav D S et al. 2017	Pune (Maharashtra)	Hospital-based study Urban Duration = Sep 2015- August 2016	DIPSI	*N* = 75 Gestational age = 24–28 weeks	7.5%
80	Anajalakshi C et al. 2009	Dharwad (Karnataka)	Hospital-based study Urban Duration = Jan to Dec 2014	DIPSI	*N* = 147 Gestational age-24 to 34 weeks	4.8%
81	Jain P et al. 2017	Nagpur (Maharashtra)	Hospital-based study Urban Duration = Nov 2013 to Oct 2015	DIPSI	N = 52 Gestational age-28 to 32 weeks	10.7%
82	Khan S et al. 2018	Western India	Hospital-based study Urban Duration = May 2012 to Apr 2014	DIPSI	N = 31 Gestational age < 20 weeks and 24–28 weeks Mean age = 24.26 ± 3.75 years	15.5%
83	Sharma N K et al. 2019	West Bengal	Hospital-based study Semi-urban Duration = June 2014 to May 2015	WHO 2013	*N* = 22 Gestational Age = 24–28 weeks Mean Age = 23.15 ± 3.9 years	11%
84	Rudra S et al. 2019	Ambala (Haryana)	Hospital-based study Rural	IADPSG	*N* = 102 Gestational Age = 24–28 weeks Mean age = 23.86 years	13.6%
85	Choudhary N et al. 2017	Jammu n Kashmir	Hospital-based study Rural Duration = July 2012 to April 2015	Carpantan and Coustan Criteria	*N* = 569	9%
86	Kalyani K R et al. 2014	Wardha (Maharashtra)	Hospital-based study Rural	WHO 1999	N = 25 Mean age = 24.16 + −3.63 years	8.33%
87	Siddique S et al. 2019 (A)	Saket (New Delhi)	Hospital-based study Urban Duration = December 2015–October 2016	ADA	N = 14 Gestational age = 2nd and 3rd Trimester Mean age- 29.719 ± 3.59 years	14%
88	Siddique S et al. 2019 (B)	Muzaffarpur (Bihar)	Hospital-based study Urban Duration = December 2015–October 2016	ADA	N = 5 Gestational age = 2nd and 3rd Trimester Mean age- 26.015 ± 5.75 years	3.07%
89	Siddique S et al. 2019 (C)	Bhilai (Chhattisgarh)	Hospital-based study Urban Duration = December 2015–October 2016	ADA	N = 7 Gestational age = 2nd and 3rd Trimester Mean age- 28.531 ± 4.51 years	10.77%
90	Dhanapal et al. 2019	Surat (Gujarat)	Hospital-based study Urban Duration = 2013 to 2016	IADPSG	N = 81 Gestational Age = all trimester	30.6%
91	Mehta et al. 1990	Baroda (Gujarat)	Hospital-based study Urban Duration = 1 year	NDDG	N = 6 Gestational Age = 24 to 28 week	4%
92	Gaana S. et al. 2020	Mysuru South India	Hospital based study Urban	DIPSI	N = 11 Gestational Age > 23 weeks	9.2%
93	Singh A et al. 2021	Lucknow	Hospital based study Urban	IADPSG	N = 35 Gestational Age = 24–28 weeks Mean Age = 25.52 ± 3.19 years	21.9%
94	Madhu SV et al. 2019	Delhi	Hospital based study Urban Duration = 2015–2017	IADPSG	N = 45 Gestational age = 24–28 weeks Mean age = 25.31 ± 3.12 years	10%
95	Balaji V et al. 2007	Chennai	Hospital based study Urban	WHO 1999	N = 86 Gestational Age = 24 to 28 weeks Mean age = 30.63 ± 4.62 years	33.37%
96	Poornima B et al. 2017	Bangalore	Hospital based study Rural Duration = October 2014 to September 2016	ADA	N = 43 Gestational Age = 24 to 28 weeks Mean age = 26 ± 3.2 years	8.5%
97	Shridevi ET AL 2015	Karnataka	Hospital based study Urban Duration = December 2013 to December 2014	DIPSI	N = 23 Gestational Age = 14 to 18 weeks	11.5%
98	Das Mukhopadhyay et al. 2020	Kolkata	Hospital-based study Urban Duration = Aug 2016 to July 2018	IADPSG	*N* = 155 Gestational Age = 24 to 28 weeks Mean age = 30.01 (3.5)	37.3%
99	Punnose J et al. 2018	Delhi	Hospital-based study Urban Duration = Jan 2006 to December 2016	NICE	*N* = 5991 Gestational Age = 24–28 weeks Mean age = 27.02 ± 3.98 yrs	16.4%
100	Garg P et al. 2017	Delhi	Hospital based study Urban Duration = Jan 2014 to June 2015	IADPSG	N = 20 Gestational Age = 24 to 28 weeks Mean age = 28 (26–28 yrs)	20%
101	Shardha SO et al. 2016	Chennai (Tamil Nadu)	Hospital based study Urban Duration = March 2013 to February 2014	DIPSI	N = 54 Gestational Age = 24 to 28 weeks Mean age = 26.08 years	22.6%
102	Jeeyasalan L et al. 2016	Vellore (Chennai)	Hospital based study Urban Duration = 15 years	?	*N* = 3902 Gestational Age = 28 to 42 weeks Mean age = 25.2 (4.2)	10.9%
103	Tellapragada C et al. 2016	Manipal (South India)	Hospital based study Urban Duration = May 2011 to April 2014	?	N = 38 Gestational Age = 20–24 weeks Mean age = 27.18 ± 3.54 years	5.2%
104	Jain R et al. 2016	Kanpur (Uttar Pradesh)	Hospital based study Urban Duration = October 1,2013 to September 31, 2014	DIPSI	*N* = 7641 Gestational Age = 24–28 weeks Mean age=	13.37%
105	Mitra S et al. 2014	Pondicherry	Hospital based study Urban Duration = August 2011 to July 2012	IADPSG	*N* = 83 Gestational Age= Mean age=	27.3%
106	Pochiraju M et al. 2014	Hyderabad	Hospital based study Urban	IADPSG	*N* = 1143 Gestational Age = 24–28 weeks Mean age = 15–49 years	17.02%
107	Nallaperumal S et 2013	Chennai	Hospital based study Urban	IADPSG	N = 599 Gestational Age = 32–34 weeks	66.6%
108	Madhavan A et al. 2008	Kottayam Kerala	Hospital based study Urban Duration = April 2005 to April 2006	ADA	N = 8 Gestational Age = 24–28 weeks	7.5%
109	Maheshwari J R et al. 1989	Mumbai	Hospital based study Urban Duration = 1st June 1987 to 31st July 1988	WHO 1999	N = 36 Gestational Age = 28–34 weeks Mean age=	10.9%
110	Singh K et al. 2020	Sikkim	Hospital based study Urban Duration = January 2019 to June2019	DIPSI	N = 24 Gestational Age = 24–28 weeks Mean age = 18–40 years	11.9%
111	Prasad DKV et al. 2022	Andhra Pradesh	Hospital based study Urban	WHO 2013	N = 8 Gestational Age = 24–28 weeks	8%
112	Swaroop N et al. 2015	Uttar Pradesh	Hospital based study Urban Duration = Jan 2014 to Jan 2015	DIPSI	N = 22 Gestational Age = 24–28 weeks Mean age = 25.46 years	9.7%
113	Dahiya K et al. 2014	Rohtak (Punjab)	Hospital based study Urban Duration = Jan 2011 to Dec 2011	DIPSI	N = 35 Gestational Age = 24–28 weeks	7%
114	Uma R et al. 2017	Chennai (Tamil Nadu)	Hospital-based study Urban	IADPSG	*N* = 247 Gestational Age = 24–28 weeks Mean age = 28.8 ± 4.4 yrs	21.9%
115	Sahu MT et al. 2007	Lucknow (Uttar Pradesh)	Hospital-based study Urban Duration = May 2005 to June 2006	ADA	N = 9 Gestational Age = 24 to 28 weeks	2.36%
116	Swami S R et al. 2008	Maharashtra (Western India)	Hospital based study Urban Duration = 2005–2007	ADA	N = 94 Gestational Age= Mean age = 25.4 years	7.7%
117	Menon U et al. 1991	Vellore (South India)	Hospital based study Urban Duration = May–August 1989	?	N = 28 Gestational Age = 30.04 ± 4.74 Mean age = 27.86 ± 4.7	11.9%

A total of 13 studies were found to report the data in separate studies which was part of a large study. The studies by Punnose J et al. 2018 [28] and Punnose J et al. 2021 [29] and Agarwal MM et al. 2018 [30] was conducted in the same population (*n* = 36,530) during the time period January 2006–December 2016 and was also reported in multiple publication. Thus, data from these studies were considered as one data and the study with the longest time period (Punnose J et al. 2018) was included in the review. Similarly, a study was conducted in the South Indian pregnant women (*n* = 304) during July 2011 to August 2012 by Nayak PK et al. 2013 [31] and Mitra S et al. 2014 [32] and was reported as separate studies. Thus, we included the Mitra S et al. 2014 with the complete data for the analysis. Similarly, a project “Women in India with GDM Strategy (WINGS)” was carried out in Tamil Nadu between January 2013 and December 2015 in Pregnant women (*n* = 1459) and were reported as two separate studies by Bhavdharini et al. (2016 and 2017). We considered them as one data and included Bhavdharini et al. 2016 in our study.

Likewise, studies, namely, Rajput R et al. 2012, Tripathi R et al. 2012, Kumar CN et al., C R et al. 2014, Bhattacharya et al. 2002, Balaji V et al. 2006, Balaji V et al. 2012, and Seshiah V et a 2007, were reported as separate studies using data from a large study and hence, were excluded from the analysis.

Five studies were added using suffix (A, B and C) as they reported the prevalence of GDM using different sub-sets of population, but were otherwise reported in the same study. Taneja et al. 2020 in Punjab used the same criteria of screening GDM in women at different gestational age (26 to 28 weeks and after 34 weeks) [33]. These were considered as 2 separate studies and labelled as Taneja (A) and Taneja (B) respectively. Similarly, a study was conducted by Siddique et al. using ADA criteria in Saket, Muzzaffarpur and Bhilai area on different subset of population [34]. These studies were also considered as three different studies and labelled as Siddique (A), (B) and (C) respectively. Also, a community based study was conducted in urban, semi-urban and rural area of Chennai city on a different sub-set of population [35]. These were considered as three different studies and labelled as Seshiah V et al. 2009 (A), (B) and (C) respectively.

A total of 19 articles utilized a combination of criteria to estimate the prevalence of GDM [36–52].

The variation in diagnostic criteria during estimation of Glucose in pregnant women pose a challenge in data extraction. Thus, the most recent and up-to-date criteria was selected in the following sequence-IADPSG/ WHO 2013 > DIPSI>WHO 1999 > ADA > NICE> Carpenter and Coustan > NDDG> O’Sullivan and Mahan’s Criteria as framed after the iterative discussion by the subject experts.

### Diagnostic criteria

A variety of diagnostic criteria were used in a total of 117 studies included in the review. (See Table 2: Different GDM Screening criteria).

**Table 2:**
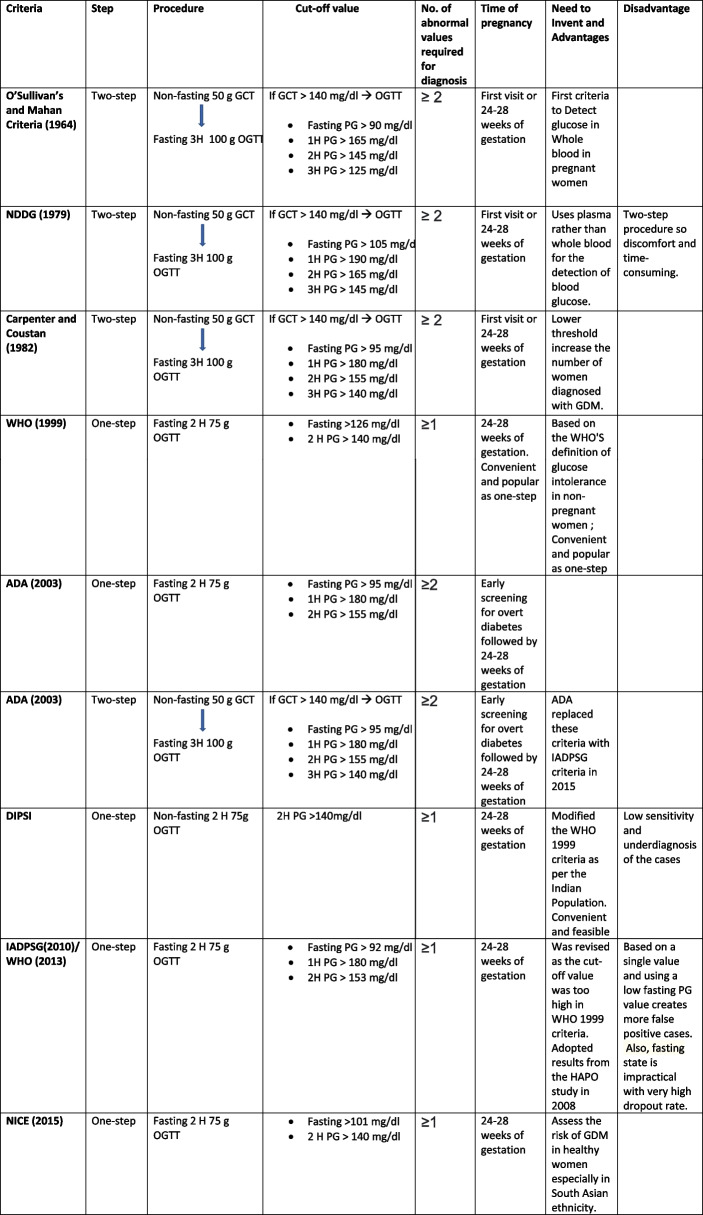
Different screening criteria used by societies for diagnosing GDM in Pregnant women

*GCT* Glucose Challenge Test: *OGTT* Oral Glucose Tolerance Test: *FPG* Fasting Plasma Glucose: *PG* Plasma Glucose: *NDDG* National Diabetes Data Group: *WHO* World Health Organization: *ADA* American Diabetes Association: *DIPSI*-Diabetes in pregnancy study group of India: *IADPSG* International Association of Diabetes Pregnancy Study Group: *NICE* National Institute for Healthcare and Excellence.

DIPSI (29 prevalence estimates) [23] was the most common diagnostic criteria used, followed by IADPSG / WHO 2013 (38 prevalence estimates) [53], WHO 1999 (24 prevalence estimates) [54], and ADA (11 prevalence estimates) [55]. Other criteria used were Carpenter and Coustan Criteria (6 prevalence estimates) [56], NDDG (1 prevalence estimate) [57], NICE (1 prevalence estimate) [58], and O’Sullivan and Mahan’s criteria (1 prevalence estimate) [59]. There was no clear description of study criteria used in 6 studies [33, 60–63].

#### Capillary versus venous blood

A total of 6 prevalence estimates used capillary blood glucose (CBG) or glucometer measurements rather than venous plasma glucose (VPG) [30, 64–68]. Three studies use capillary blood followed by venous blood glucose estimation [12, 48, 69]. In 3 studies, a comparative assessment of capillary and venous blood glucose estimation was done on the prevalence of the GDM in the pregnant women [70–72].

#### Two-step versus one-step procedure

A total of 93 studies (*n* = 93) uses one-step procedure to estimate the prevalence of GDM, whereas, only 19 studies (*n* = 19) used two-step procedure for the diagnosis of the GDM in the study population. There was no clear description of study criteria used in 5 studies.

### Risk of Bias

**Fig. 2:**
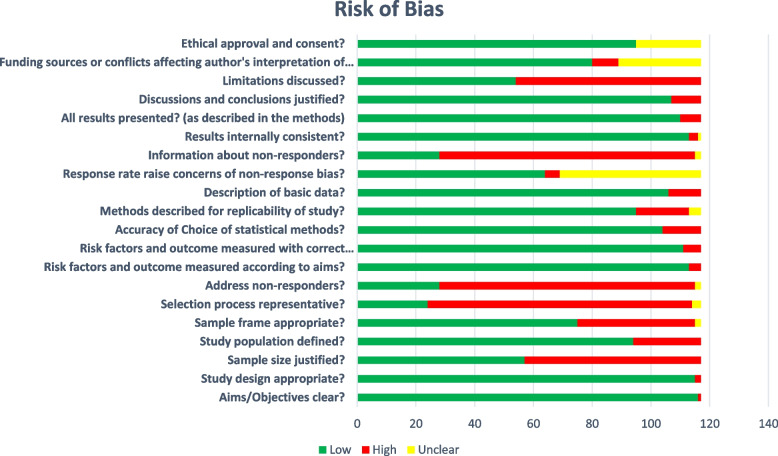
Risk of Bias Assessment

We assessed the Risk of Bias using the AXIS tool [26]. Overall, 117 studies were included in the Risk of Bias assessment using the AXIS tool. A horizontal bar graph showing the Risk of bias tool result for each component is given in Fig. 2 Risk of Bias.

Majority of the study components revealed a low risk of bias namely, objective of the study, appropriateness of the study design, study population defined, appropriateness of sample frame, risk factors measured according to the objectives and with the appropriate study tool, accuracy of choice of statistical methods, measures of replicability of the study, description of the basic data, results internally consistent, all results presented and justification of discussion and conclusion.

There was no clear description of response rate bias in 48 studies. Also, there was no description of Ethical consent in 22 studies. Only 9 studies reported funding, but there was no clarity of 28 studies on their funding sources keeping them in unclear risk of bias.

A high risk of bias was revealed in the sample size justification in 57 studies. Further, the results from 90 studies lacks generalizability to the general population marking them with high risk of bias. There was no description about non-responders and their information in 87 studies revealing the high risk of bias. Many studies (*n* = 63) which did not discuss their limitations were categorized as having high risk of bias.

### Prevalence estimates of GDM in pregnant women in India

The final 117 studies were used for prevalence estimates of GDM in pregnant population in India. A total of 106 studies were conducted in a hospital-based setting and 11 were community-based studies.

**Fig. 3:**
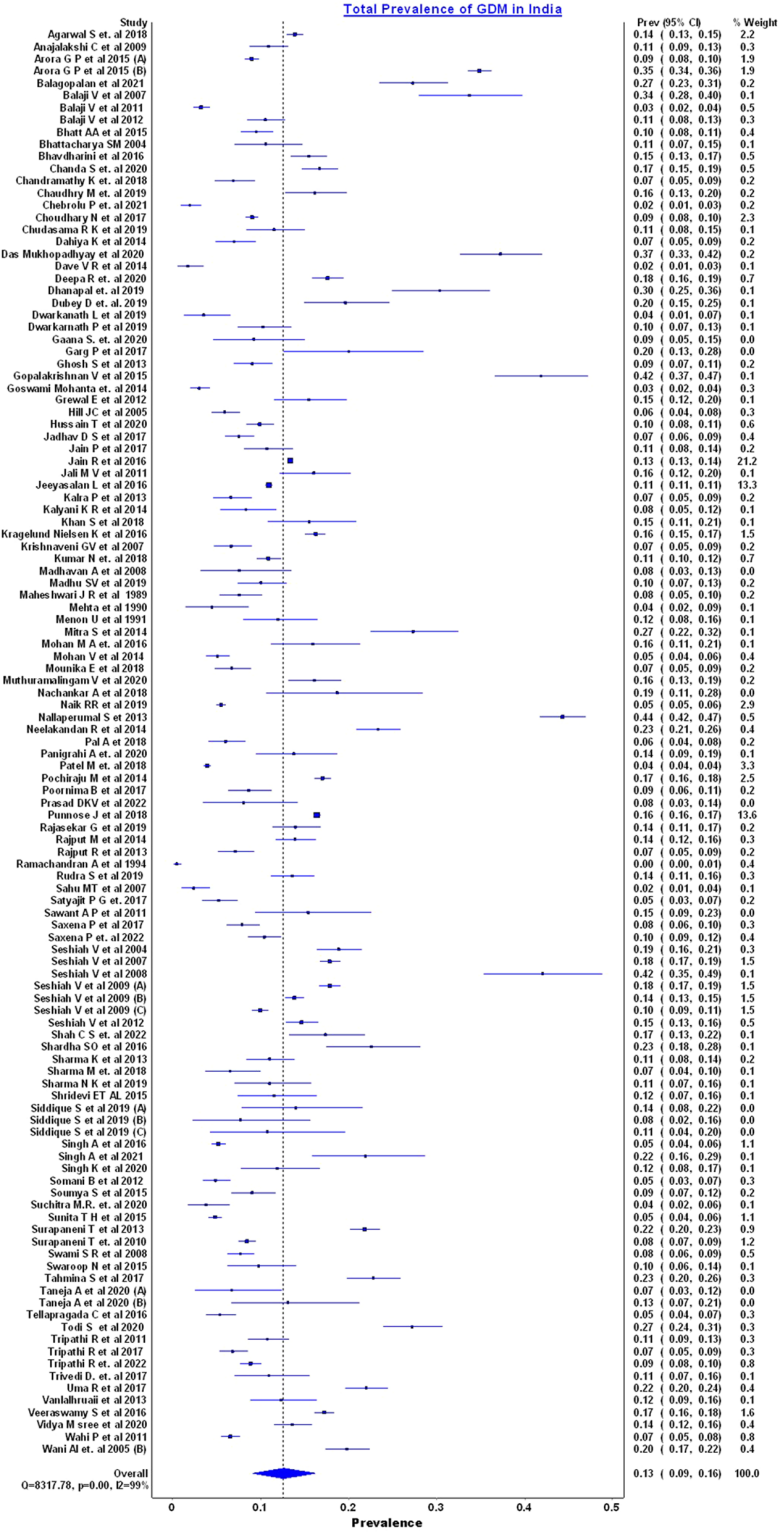
Forest Plot depicting the pooled prevalence of GDM in India

We found a pooled estimate (with an Inverse square heterogeneity model) of the prevalence of overall GDM in pregnant women was 13% [95% CI, 9–16%, *n* = 117 studies] with the heterogeneity of the studies high at 99% which restricts the generalizability of the findings **(**Fig. 3 Forest Plot depicting the pooled prevalence of GDM in India) The possible reasons could be studies varied widely in population type, geography, as well as the diagnostic method used. (Table 3 Sub group Analysis) The publication date of the studies ranged from 1989 to 2022.

- ***Geographical Zones***

**Table 3:** Subgroup analysis of overall Gestational Diabetes Mellitus estimates

Subgroup categories	Number of studies	ES	95% CI	I^2^(%)	Cochrane Q
**Geographical Zones**					
*North*	31	16.0	9.8-22.8	98.8%	2541.5
*South*	47	12.6	7.8-17.8	98.3%	2911.9
*Central*	13	12.0	4.3-21.1	99.2%	1692.6
*West*	17	7.0	3.3-11.2	93.8%	259.7
*North-Eastern*	9	11.5	5.3-18.4	97.3%	304.7
**Population**					
*Urban*	92	12.5	8.7-16.4	98.5%	6152.6
*Semi-urban*	8	16.0	7.2-25.9	99.4%	1522.9
*Rural*	17	9.7	6.4-13.3	95.5%	357.2
**Criteria**^**a**^					
*DIPSI*	29	11.7	5.6-18.5	97.9%	1394.9
*WHO 1999*	24	12.5	9.2-15.9	97.4%	917.6
*IADPSG / WHO 2013*	38	16.6	12.0-22.0	98.8%	3296.0
*ADA*	11	7.1	4.2-10.4	86.2%	72.8
*Carpenter –Coustan*	6	7.3	4.2-10.6	95.1%	103.8
*NICE*	1	16.4	16.0-16.8		
*NDDG*	1	4.4	1.5-8.7		
*O’Sullivan Criteria*	1	4.0	1.0-10.0		

^a^No description of Diagnostic criteria used in 6 studies

India has a union of 28 states and 8 Union territories, divided as “North,” “South,” “East,” “Central” or “West” based on the Inter-state council secretariat classification of geographic regions of India [73]. Therefore, region-wise subgroup analysis was also conducted to get estimates of the prevalence of GDM. North region includes Haryana, Himachal Pradesh, Punjab, Delhi, Chandigarh, Uttarakhand, Jammu and Kashmir and Ladakh. States like Gujarat, Rajasthan, Maharashtra, Goa, Daman and Diu and Dadara and Nagar Haveli comprises West Region of India. South India includes Andhra Pradesh, Karnataka, Kerala, Tamil Nadu, Telangana, Andaman and Nicobar Islands, Lakshadweep and Puducherry. East and North-eastern states are Bihar, Jharkhand, Odisha, West Bengal, Arunachal Pradesh, Sikkim, Mizoram, Assam, Meghalaya, Manipur, Nagaland and Tripura. Central Zone of India includes Chhattisgarh, Uttar Pradesh and Madhya Pradesh.

**Fig. 4:**
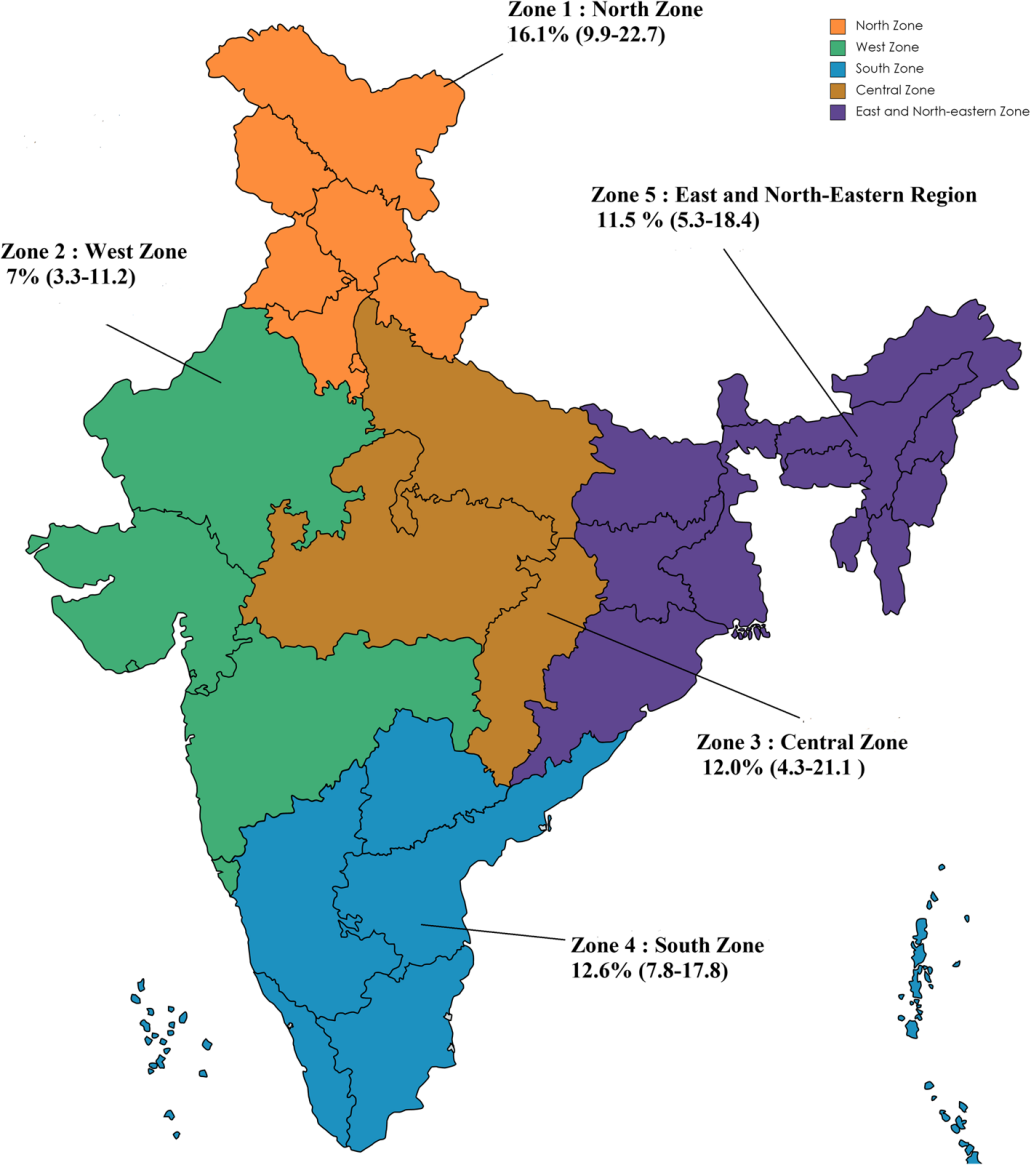
Map of India showing prevalence of GDM in 5 different zones of India

The prevalence of GDM varies across the 5 zones of India. The highest prevalence of GDM was found in North region followed by South India. Areas of low prevalence include West, Central and Eastern zone of India. One of the confounding factors behind low prevalence could be lesser studies conducted in these zones to estimate the prevalence. (Fig. 4 Map of India showing the prevalence of GDM in 5 different zones of India).

The pooled prevalence of GDM in ***North Zone*** was found to be 16.1% [95% CI, 9.9–22.7, I^2^ = 98.9%, *n* = 31 studies]. The maximum weightage (36.53) was by a study from Punnose J et al. conducted in 2018 [28].

Similarly, the pooled prevalence of GDM in ***West Zone*** was found to be 7% [95% CI, 3.3–11.2, I^2^ = 98.9%, *n* = 17 studies]. The maximum weightage (50.24) was by a study from Naik RR et al. 2019 [74].

In ***Central Zone***, the pooled prevalence of GDM was found to be 12.0% [95% CI, 4.3–21.1, I^2^ = 99.29%, *n* = 13 studies]. A study by Jain R et al. conducted in 2016 has a maximum weightage of 66.55 [75].

The pooled prevalence of GDM in ***South Zone*** was 12.6% [95% CI, 7.8–17.8, I^2^ = 98.38%, *n* = 47 studies]. The maximum weightage was held with study by Jeeyasalan L et al. conducted in 2016 [63].

In ***East and North-eastern Region***, the pooled prevalence of 11.5% was found. [95% CI, 5.3–18.4, I^2^ = 97.34%, *n* = 9 studies]. The maximum weightage (27.27) by a study done by Hussain et al. in 2020.) [76].

b) ***Urban*** **versus** ***Rural Studies***

A total of 92 studies were conducted in urban areas, 8 studies in semi-urban areas and 17 studies in rural areas. The pooled prevalence in the rural population was 10.0% [6.0–13.0%, I^2^_=_96%, *n* = 10 studies], whereas, the pooled prevalence of 12.0% [9.0–16.0%, I^2^ = 99%, *n* = 88 studies] was found in the urban population. A study conducted by Seshiah V et al. in 2009 included the study participants from urban, semi-urban and rural areas of Tamil Nadu [35].

c) ***Diagnostic and Screening criteria***

With the subgroup-analysis using diagnostic criteria, the prevalence of GDM using WHO 1999 criteria was 12.0% (9.0–16.0%), I^2^_=_97% studies, *n* = 57 studies] which was slightly less than the prevalence of GDM with DIPSI criteria [23] 13.0% [3.0–24.0%, I^2^_=_99%, *n* = 29 studies] The IADPSG/ WHO 2013 criteria detected a higher prevalence of GDM as 17.0% [12.0–22.0%, I^2^ = 99%, *n* = 38 studies], while, ADA criteria pooled a lower prevalence of 7.0 [4.0–10.0%, I^2^ = 86%, *n* = 11 studies]. There was prevalence range of 13.0% [3.0–24.0%, I_2_ = 99%, n = 9 studies] was using other criteria like C&C criteria, NICE, NDDG and O′ Sullivan Criteria.

#### Small study effects

**Fig. 5:**
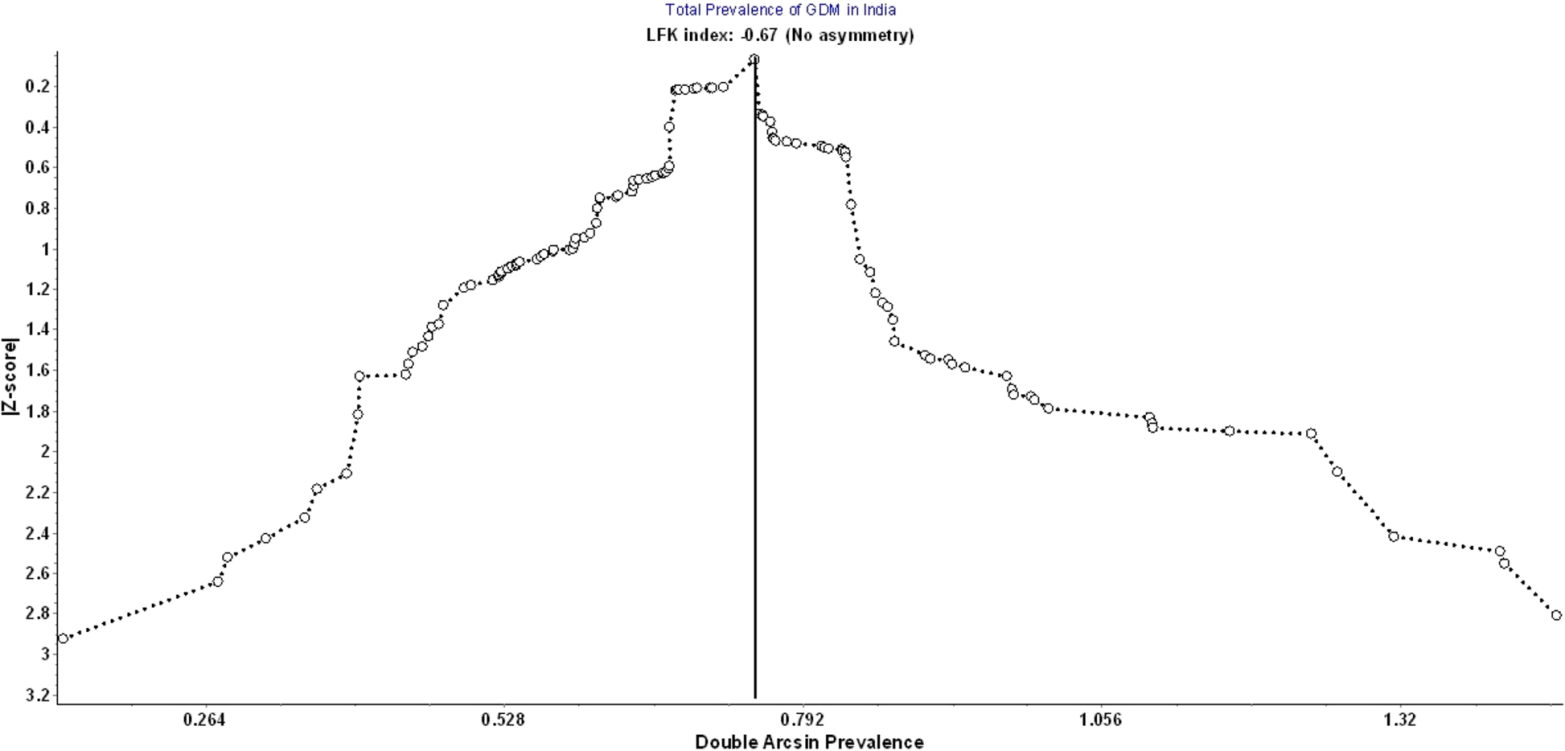
DOI plot for publication bias

We evaluated the small study effects like publication bias using the DOI plot and LFK index. There was no asymmetry in the National pooled estimate [LFK index = − 0.67] and Zonal estimate except for the North zone and West zone. (See Fig. 5: DOI Plot for Publication bias).

## Discussion

Plethora of studies discussing the GDM prevalence in India are published, but there is a scarcity of studies discussing the regional estimates of GDM prevalence in India. A systematic review and meta-analysis conducted by Katherine T Li et al. quantitatively examined the prevalence of GDM across India based on 64 studies up to the year 2016 and explored the prevalence of GDM which ranged 0 to 41.9% [77].

This systematic review and meta-analysis included 110 studies reporting the prevalence of GDM ranging from 9 to 16% in pregnant women in India. We found a pooled estimate (with an Inverse square heterogeneity model) of the prevalence of overall GDM in pregnant women was 13% [95% CI, 9–16%] with the heterogeneity of the studies high at I_2_ = 99%. The possible reasons behind this heterogeneity could be studies varying widely in population type, geography, study duration and the diagnostic method used. Our study also highlighted the discrepancy in prevalence estimates due to different screening criteria, gestational age of screening, capillary versus venous blood estimation and one-step versus two-step procedure used for diagnosing GDM.

### Which diagnostic criteria is suitable for Indian pregnant women?

The most commonly used criteria were DIPSI followed by IADPSG/WHO 2013 and WHO 1999. With descriptive analysis, we found that the WHO 1999 criteria detected a high prevalence of GDM as compared to IADPSG and DIPSI which almost detected the pooled prevalence of 12–13%.

Das Mukhopadhyay et al. did not find any significant difference between the prevalence rates of GDM among DIPSI and IADPSG criteria [52]. But he concluded that DIPSI being simple in execution and patient friendly is close to the international consensus. In a study by Singh et al. in 2021, it was observed that DIPSI was only 37.1% sensitive as compared to IADPSG criteria [51]. Contrary to these findings, Seshiah et al. found a high concordance between DIPSI and IADPSG criteria [78]. The low sensitivity of DIPSI has been reported by studies such as Mohan et al.2014 [41]. and Herath et al. [79]. Sensitivity of DIPSI is quite low, hence to be used as screening and diagnostic tool at the same time is still questionable. This is the dire requirement of our country to have a better sensitive method for diagnosing GDM so that patients do not escape diagnosis (false-negatives cases) detected by DIPSI which later on crunch out the health system.

Indeed, in 2013, the WHO embraced the IADPSG criteria, replacing the earlier 1999 criteria. The DIPSI criteria were formulated based on the 2-hour post-glucose (PG) values of the WHO 1999 criteria, primarily focusing on the simplicity of assessing the 2-hour PG value independently. It’s important to note that the Fasting Plasma Glucose (FPG) parameter from the WHO 1999 criteria is considered outdated now, indicative of diabetes [53].

Further, IADPSG recommendation necessarily requires estimation of plasma glucose in three blood samples after administrating 75 g oral glucose load. Pregnant women resent this procedure, as they are pricked three times and feel too much of blood is drawn. Whereas, DIPSI criterion requires one blood sample drawn at 2-h for estimating the plasma glucose Future studies should compare the outcomes of the GDM cases diagnosed by different criteria as this would provide the final answer as to which criteria is more suitable for Indians.

### Does sensitivity and Specifity of the diagnostic test matters?

A study by Mohan V et al. in 2014 compared the IADPSG, DIPSI and WHO 1999 criteria shows that the non-fasting OGTT has poor sensitivity compared to both the WHO 1999 criteria (27.7%) and the IADPSG criteria (22.6%) [41]. Thus, the current DIPSI guidelines of doing a single-step non-fasting OGTT using the 2-h VPG cut point of 140 mg/dl (7.8 mmol/l) to diagnose GDM would miss 72.3% of women with GDM diagnosed by the WHO 1999 criteria and 77.4% of women with GDM diagnosed by the IADPSG criteria. Similarly, a study by Tripathi R et al. 2017, a two-hour 75 g non-fasting DIPSI test was done and followed by OGTT [40]. Using OGTT as per the WHO 2013 /IADPSG criteria as gold standard, the sensitivity of 75 g non-fasting test was low. With this low sensitivity, about one quarter of women with GDM were missed. Missing such a large number is not acceptable for a diagnostic test, especially as GDM is associated with both maternal and perinatal complications. On contrary, in the study population, Seshiah V 2012, utilized both DIPSI and IADPSG criteria to ascertain the prevalence of GDM, which were 13.4 and 14.6% respectively [43].

### Which is appropriate- early screening or risk-based screening?

There is a debate regarding the timing of screening for GDM, whether it should be done during the first prenatal visit or during the recommended period of 24–28 weeks of gestation. On the question of when to screen for GDM, a descriptive analysis by Li et al. 2018 showed that a substantial percentage of patients (11.4–60% of GDM cases) develop GDM in the first trimester, but that a similarly large percentage of patients (16–40% of GDM cases) are missed at the first visit [77]. Conducting the screening at later stages of pregnancy is linked to increased risks of maternal and perinatal morbidity and mortality. Many studies on GDM also suggest that early screening and dietary control of GDM can promote the curtailment of maternal and perinatal morbidities [80, 81]. Additionally, Raets et al. demonstrated that there is need for clear guidelines and criteria concerning early screening for GDM [82]. In line with the Flemish consensus of 2019 on screening for GDM, this review recommend to universally screen for diabetes in early pregnancy [83].

Therefore, the review findings indicates an early screening with an OGTT test at 24 weeks coupled with diet counselling and postpartum testing in pregnant women can improve perinatal outcomes [75]. However, this may not be a logistically feasible or cost-effective strategy for all patients, and screening may need to be risk-stratified in Low or Middle Income Country (LMIC).

### How should pregnant women come for GDM screening- fasting or non-fasting?

In their study, Supraneni et al. conducted a comparative analysis of the diagnostic effectiveness of different fasting plasma glucose levels and the one-hour 75 g OGTT in diagnosing GDM [84]. The study found that fasting plasma glucose levels above 92 mg/dL exhibited better diagnostic effectiveness, but there was no significant difference when compared to the results obtained from the one-hour 75 g OGTT in distinguishing between pregnant women with and without GDM.

Additionally, the researchers observed that utilizing the International Association of Diabetes and Pregnancy Study Groups (IADPSG) cutoffs for fasting and one-hour 75 g OGTT demonstrated good diagnostic properties in the study population. By implementing an exit strategy based on a positive result at either the fasting or one-hour mark, it was estimated that the need for further testing could potentially be reduced in approximately one in five pregnant women. However, accessing antenatal care in a fasting state posed challenges in rural settings, as highlighted in a 2014 study by Mohan et al. [41]. On the other hand, the DIPSI (Diabetes in Pregnancy Study Group India) guidelines suggest that the GDM test can be conducted at any time during pregnancy, regardless of food intake [85]. Nevertheless, the DIPSI approach faces difficulties in effectively screening pregnant women for GDM due to low sensitivity and underdiagnosis [86].

Based on the findings of the review, it is clear that a significant need exists for well-designed and unified programs aimed at effectively managing GDM cases. Implementing such programs would be instrumental in reducing the escalating burden of diabetes in India.

### Capillary versus venous blood – does it affect estimation?

There is contradictory evidence reporting varying results and conclusions regarding the accuracy and agreement between blood glucose estimation using venous plasma glucose (VPG) and capillary blood glucose (CBG) methods for diagnosing GDM.

The study by Balaji V in 2012 involving a significant number of cohorts indicated that the Accu-Chek glucometer, a CBG measurement device, provided accurate results that aligned well with laboratory measurements of VPG [72]. Similarly, another study reported that CBG values provided the closest approximation to VPG values in healthy individuals without diabetes or GDM [66]. On the other hand, Jadhav DS conducted a hospital-based clinical study in 2017 comparing VPG and CBG estimation using a glucometer based on the DIPSI criteria found a satisfactory level of agreement between the two methods with equal sensitivity. Additionally, the CBG estimation by glucometer demonstrated a small number of false positive cases due to its high specificity (99.46%) [70].

Indeed, it is interesting to note that in some studies, the capillary blood glucose (CBG) and venous plasma glucose (VPG) values were found to be similar at 1 hour (9.9 mmol/L vs. 9.6 mmol/L) and 2 hours (7.9 mmol/L vs. 7.7 mmol/L) after the glucose load [87]. These findings suggest a fair agreement between CBG and VPG measurements during the 2-hour OGTT test for (GDM.

However, it is worth mentioning that other studies have reported a slight difference between VPG and CBG values, ranging from 0.28 to 0.5 mmol/L (5–9 mg/dL) specifically at the 2-hour mark, although the difference is relatively small [88]. These discrepancies in findings may be attributed to several factors, including the specific population under study, the glucose measurement methods used, and the performance characteristics of the glucose measurement devices employed [89]. The accuracy and agreement between CBG and VPG measurements can vary across different studies and settings.

A recent study by VidyaM Sree et al. demonstrated an excellent diagnostic accuracy (99.77%) of CBG estimation using a one-step OGTT based on DIPSI criteria for GDM in an Indian population. This study highlighted the feasibility and reliability of capillary blood estimation for GDM screening, particularly in countries with limited resources [71].

This review led to the conclusion that capillary blood estimation is a feasible and reliable method for screening GDM In countries with limited resources as this approach requires less technically trained manpower and equipment. It is important for further research to explore and address these differences in order to establish standardized guidelines and protocols for the diagnosis and management of GDM, particularly in terms of blood glucose estimation methods.

### Cost-effectiveness and feasibility- what should be preferred?

The prevalence of GDM varies across different states in India, highlighting the country’s diversity. Even if a universally applicable, feasible, diagnostically accurate, and cost-effective test for GDM is discovered, the gravity of the problem remains consistent.

Supraneni et al. discovered in his study that the IADPSG criteria have good specificity, positive likelihood ratio and post-test probabilities for GDM in their study population [87]. However, the cost involved for performing IADPSG recommended procedure is high, as this procedure requires three blood tests compared to one blood test of DIPSI.

“DIPSI as one-step screening and diagnostic procedure for assessing GDM in pregnant women which is less time-consuming, economical and feasible” as stated by Mounika E et al. in her study conducted in south Indian Population [47]. But, the large extent of false negatives is a major limitation of DIPSI test which cannot be overlooked. Swaroop N et al. used one-step DIPSI criteria in his study and found it to be effective but larger studies are required to further validate its importance [90].

Thus, this review suggests that ideally, and whenever feasible, a single-step 75-g OGTT using the IADPSG criteria should be done in the fasting state as this is the accepted criteria worldwide and would help to bring about international standardization. However, in countries with less resources, DIPSI criteria may be used as a backup option in certain situations where it would be cost-effective without compromising the clinical equipoise: (a) inaccessible areas where pregnant females are not able to visit healthcare facility in fasting state in morning (b) epidemiological studies where fasting sample is unavailable (c) where OGTT is not feasible in some pregnant females due to certain specified reason.

### Strength of the review

Our review raises a valid point regarding the challenges of implementing a universal screening program for GDM in India. We have taken into account unpublished literature from the Indian database ShodhGanga to gather comprehensive information about the current scenario of GDM in different zones of India. We have made efforts to contact authors to obtain full-text articles or any necessary information for our analysis, ensuring maximum data inclusion.

The review highlights the need for policymakers to reach a consensus on a universal screening test for diagnosing GDM in pregnant women, considering various key factors. These factors include the variation in diagnostic criteria, such as fasting or non-fasting, one-step or two-step approaches, and the use of capillary or venous blood estimation. Additionally, the review considers the sensitivity and specificity of the diagnostic test, the cost-effectiveness of the screening method, and its feasibility in real-world settings.

We also conducted an analysis to assess publication bias. However, since we have included prevalence studies, the results can be generalized to the population regardless of any bias. Furthermore, we performed a sub-group analysis to provide an overview of the current pooled prevalence of GDM in different geographic zones of India.

The authors suggest that implementing a uniform approach nationwide may not be practical. Instead, they propose adopting a more focused and region-specific strategy to maximize resources and efficiently detect and address cases of GDM.

Overall, our review aims to provide evidence-based insights and encourage policymakers to develop consensus guidelines for GDM screening in India. By considering the diverse factors and conducting thorough analyses, we hope to contribute to the formulation of effective strategies for GDM diagnosis and management across the country.

### Limitations

Although we comprehensively searched four databases, we may have included a few more databases to include more GDM-related studies. Further, analyzing the risk factors involved in the prevalence of GDM was not in the scope of our review. Further, some studies did not provide detailed information about their population type, their GDM screening methods, trimester or the distribution between multiple different screening methods that were used. It is imperative to acknowledge the absence of a standardized screening strategy, which introduces a significant limitation to our analysis. Furthermore, we recognize the potential influence of evolving diagnostic criteria on variations in GDM prevalence. To address this concern, it would be beneficial to incorporate a comparative analysis of GDM prevalence across different regions, focusing on studies that employ consistent diagnostic criteria such as DIPSI or IADPSG (WHO 2013). Additionally, we acknowledge that differences in prevalence may be attributed to assessments conducted in distinct time periods. As a means to enhance the comprehensiveness of our review, we highlight the importance of exploring studies that specifically examine trends in GDM within a given population in India over time.

## Conclusion

This review emphasizes the growing concern of GDM as a public health issue, particularly in resource-constrained settings like India, where the prevalence of GDM varies significantly among states. Numerous studies conducted in India have revealed poor agreement among existing diagnostic criteria for GDM. To enable prompt diagnosis and enhance the management of GDM in India, it is crucial to incorporate a diagnostic tool that is feasible, cost-effective, and reliable. Such a tool should seamlessly integrate with the existing public healthcare system and benefit the target population. Large-scale population-based studies are necessary to address the conflicts in GDM diagnosis and provide evidence-based criteria that are applicable to the Indian population. By tailoring the screening program based on regional variations, healthcare authorities can better allocate resources and implement interventions focused on areas with higher GDM prevalence or other risk factors.

### Supplementary Information

**Additional file 1.**

## Data Availability

Available from the corresponding author on reasonable request.

## Abbreviations

GDM: Gestational Diabetes Mellitus
DIPSI: Diabetes in Pregnancy Study group of India
IADPSG: International Association of Diabetes and Pregnancy Study Group
HAPO: Hyperglycemia and Adverse pregnancy outcomes
FOGSI: Federation of Obstetric and Gynecological Societies of India
LMIC: Low-or-Middle Income Country
OGTT: Oral Glucose Challenge Test
