# Supplementary material for: National and regional prevalence of gestational diabetes mellitus in India: a systematic review and Meta-analysis
Source: BMC Public Health. 2024 Feb 20;24:527. doi: 10.1186/s12889-024-18024-9 (PMC10877914; doi:10.1186/s12889-024-18024-9)
Supplement: Supplementary file 1 — Additional file 1. [file 12889_2024_18024_MOESM1_ESM.pdf]

<u>Additional File 1</u>

Search strategy used for different databases are listed below: -

PubMed:
((Gestational Diabetes Mellitus) OR (GDM)) OR (Gestational diabet*)) OR (pregnancy induced diabet*)) OR (hyperglycemia during pregnancy)) OR (impaired glucose tolerance)) OR (gestational hyperglycemia)) OR (glucose intolerance in pregnancy)) OR (DIABET*)) OR (Gestational glucose intolerance)) OR (postpartum diabetes))

AND ((pregnant woman)) OR (pregnancy complications)) OR (pregnancy)) OR (gestation*)) OR (gravid*))

AND ((prevalence)) OR (epidemi*))

AND (India)) OR (India*)) OR (Andhra Pradesh)) OR (Arunachal Pradesh)) OR (Assam)) OR (Bihar)) OR (Chhattisgarh)) OR (Goa)) OR (Gujarat)) OR (Haryana)) OR (Himachal Pradesh)) OR (Jharkhand)) OR (Karnataka)) OR (Kerala)) OR (Madhya Pradesh)) OR (Maharashtra)) OR (Manipur)) OR (Meghalaya)) OR (Mizoram)) OR (Nagaland)) OR (Odisha) OR (Punjab)) OR (Rajasthan)) OR (Sikkim)) OR (Tamil Nadu)) OR (Telangana)) OR (Tripura)) OR (Uttarakhand)) OR (Uttar Pradesh)) OR (West Bengal)) OR (Andaman and Nicobar Islands)) OR (Chandigarh)) OR (Dadara and Nagar Haveli)) OR (Daman and Diu)) OR (Delhi)) OR (Jammu and Kashmir)) OR (Ladakh)) OR (Lakshadweep)) OR (Puducherry))

AND ((ffrft[Filter]) AND (fha[Filter]) AND (booksdocs[Filter] AND observationalstudy[Filter]) AND (fft[Filter]) AND (humans[Filter]) AND (Medline[Filter]))

Filters: Abstract, Free full text, Full text, Books and Documents, Observational Study, Humans, MEDLINE

Scopus:
(TITLE-ABS-KEY ("Pregnant Women" OR "Gravidity" OR "Pregnancy" OR "Pregnant")

AND TITLE-ABS-KEY ("Gestational Diabetes Mellitus" OR "GDM" OR "impaired glucose tolerance" OR "gestational hyperglycemia" OR "hyperglycemia in pregnancy" OR "glucose intolerance")

AND ("prevalence")

AND TITLE-ABS-KEY ("India" OR "Andhra Pradesh" OR "Arunachal Pradesh" OR "Assam" OR "Bihar" OR "Chhattisgarh" OR "Goa" OR "Gujarat" OR "Haryana" OR "Himachal Pradesh" OR "Jharkhand" OR "Karnataka" OR "Kerala" OR "Madhya Pradesh" OR "Maharashtra" OR "Manipur" OR "Meghalaya" OR "Mizoram" OR "Nagaland" OR "Odisha" OR "Punjab" OR "Rajasthan" OR "Sikkim" OR "Tamil Nadu" OR "Telangana" OR "Tripura" OR "Uttarakhand" OR "Uttar Pradesh" OR "West Bengal" OR "Andaman

Nicobar Islands" OR "Chandigarh" OR "Dadara Nagar Haveli" OR "Daman Diu" OR "Delhi" OR "Jammu Kashmir" OR "Ladakh" OR "Lakshadweep" OR "Puducherry"))

Google Scholar:
allinTitle: Gestational+Diabetes+Mellitus+India

ShodhGanga:
Gestational Diabetes Mellitus + Prevalence.
